# Carbon Use Efficiency and Its Temperature Sensitivity Covary in Soil Bacteria

**DOI:** 10.1128/mBio.02293-19

**Published:** 2020-01-21

**Authors:** Grace Pold, Luiz A. Domeignoz-Horta, Eric W. Morrison, Serita D. Frey, Seeta A. Sistla, Kristen M. DeAngelis

**Affiliations:** a Graduate Program in Organismic and Evolutionary Biology, University of Massachusetts Amherst, Amherst, Massachusetts, USA; b Department of Microbiology, University of Massachusetts Amherst, Amherst, Massachusetts, USA; c Department of Natural Resources and the Environment, University of New Hampshire, Durham, New Hampshire, USA; d Natural Resources Management and Environmental Sciences, California Polytechnic State University, San Luis Obispo, California, USA; Oregon State University

**Keywords:** carbon use efficiency, comparative genomics, growth strategy, physiology, soil bacteria

## Abstract

The strategy that microbial decomposers take with respect to using substrate for growth versus maintenance is one essential biological determinant of the propensity of carbon to remain in soil. To quantify the environmental sensitivity of this key physiological trade-off, we characterized the carbon use efficiency (CUE) of 23 soil bacterial isolates across seven phyla at three temperatures and with up to four substrates. Temperature altered CUE in both an isolate-specific manner and a substrate-specific manner. We searched for genes correlated with the temperature sensitivity of CUE on glucose and deemed those functional genes which were similarly correlated with CUE on other substrates to be validated as markers of CUE. Ultimately, we did not identify any such robust functional gene markers of CUE or its temperature sensitivity. However, we found a positive correlation between rRNA operon copy number and CUE, opposite what was expected. We also found that inefficient taxa increased their CUE with temperature, while those with high CUE showed a decrease in CUE with temperature. Together, our results indicate that CUE is a flexible parameter within bacterial taxa and that the temperature sensitivity of CUE is better explained by observed physiology than by genomic composition across diverse taxa. We conclude that the bacterial CUE response to temperature and substrate is more variable than previously thought.

## Abstract (precis)

Soil microbes respond to environmental change by altering how they allocate carbon to growth versus respiration—or carbon use efficiency (CUE). Ecosystem and Earth System models, used to project how global soil C stocks will continue to respond to the climate crisis, often assume that microbes respond homogeneously to changes in the environment. In this study, we quantified how CUE varies with changes in temperature and substrate quality in soil bacteria and evaluated why CUE characteristics may differ between bacterial isolates and in response to altered growth conditions. We found that bacterial taxa capable of rapid growth were more efficient than those limited to slow growth and that taxa with high CUE were more likely to become less efficient at higher temperatures than those that were less efficient to begin with. Together, our results support the idea that the CUE temperature response is constrained by both growth rate and CUE and that this partly explains how bacteria acclimate to a warming world.

## INTRODUCTION

Optimum allocation of carbon (C) to growth versus maintenance is central to the success of microorganisms. This “carbon use efficiency” (CUE) is the outcome of a complex interplay between biotic and abiotic factors which shape whether organisms are able to thrive or just survive in their environment. In turn, how CUE responds to a changing world is expected to have far-reaching implications for the ability of soils to globally maintain vital ecosystem services such as carbon retention.

Of particular pertinence is projecting how elevated temperatures are affecting microbial physiology under conditions of climate change. In ecosystem and Earth System models, CUE is typically parameterized either to be unaffected by warming or to exhibit a homogeneous community-level decrease (1–3). In response to short-term increases in temperature, community-level CUE can increase (4, 5), decrease (4, 6), or remain unaffected (4, 6–8), with no clear explanation as to why these temperature responses differ (9). Organism-level CUE decreases in response to increasing temperature when respiration increases more than growth; this accelerated respiration can be caused by increased protein turnover (10), changes in membrane fluidity (11), or the loss of energy-conserving sites in the electron transport chain (12). On the other hand, CUE is expected to increase with temperature if maintenance costs are independent of growth rate and if growth rate increases with temperature (13). In addition to these direct impacts of elevated temperature, longer-term changes in temperature may additionally play out to indirectly impact CUE through chronic warming-induced changes in the environment.

The response of CUE to long-term warming is variable (6, 14). Chronic warming can impact the quantity and quality of substrates available through their differential production and consumption (6, 15). For example, warming has been observed to increase both the quantity of C released by roots into the soil (16) and the relative contribution of phenolic compounds (17), such that microbial activity was found previously to be stimulated by warming in the rhizosphere (16). In other instances, however, warming may reduce the amount of C that plants allocate belowground (18), or plant inputs may not keep pace with increased microbial demand at higher temperatures (19). In such cases, labile compounds are preferentially lost from soil and the available substrates show signatures of a later state of decay (15). All said, these indirect effects of temperature on CUE via changes in substrate quality may be as important as—if not more important than—its direct effects (6). This is in part because intrinsic differences in the oxidation states of substrates set an upper limit on how efficiently they are anabolized (20); energy must be invested to enable the oxidation state of C in organic acids to achieve that of the cell but not for more highly reduced lipids. Substrates also differ in their extracellular processing and uptake costs, which impacts the maximum potential yield of a substrate pool (1, 6). Furthermore, the presence of alternative metabolic pathways for processing the same substrate means that microbes may differ in how much energy they can capture (21). Finally, bacteria may switch between metabolic pathways depending on temperature or substrate availability (22), opening the possibility of gene-substrate interactions affecting how CUE responds to temperature. In addition to the differences in efficiency due to substrate quality, bacteria may differ in their maximum possible efficiency (23). As such, community-level differences in CUE temperature response may be underlain by both shifts in who or what is active and the direct physiological effects of warming on a fixed community.

It has long been assumed that a trade-off exists between how fast an organism can grow and growth efficiency for a given amount of substrate—the so-called growth rate-yield trade-off (24). Bacteria with more rRNA operons are able to sustain a higher maximum growth rate (25) but also appear to grow less efficiently than those with fewer copies (23). This is proposed to be a consequence of the high energetic costs of building and running translational machinery (26), suggesting that oligotrophic bacteria are more efficient than copiotrophs. Bacteria capable of producing large amounts of extracellular enzymes (EEs) may also be less efficient than those with more-limited extracellular enzyme production capacity because substantial energy investment is required to polymerize amino acids under aerobic conditions (27, 28). The ability to produce copious and diverse extracellular enzymes may also be indicative of reduced temperature sensitivity of these taxa, however, as bacteria with diverse metabolic potentials may be better able to tune which pathways or enzymes they use in order to maintain efficiency even as the environment around them changes (29, 30). As such, genomic traits such as rRNA operon copy number (rrN) or extracellular enzyme gene density may serve as “genomic markers” of bacterial CUE and its temperature sensitivity. However, empirical support is equivocal for the growth rate-yield (23–25, 31, 32) and enzyme cost (28, 33) hypotheses, and a number of questions regarding the genomic basis of efficiency remain. Specifically, are there genomic markers of CUE which are consistent across phylogenetically diverse soil bacteria? And can the genomic repertoire of soil bacteria be used to infer the temperature responsiveness of CUE?

We sought to first quantify how soil bacterial CUE varies in its response to shifts in temperature and substrate and then to determine whether these shifts can be predicted based on genome composition. Because it is a complex metabolic trait, we posited that CUE and its temperature sensitivity (Q10) would be highly variable across taxa but would be more similar in closely related bacteria (34). Furthermore, we posited that CUE would be negatively correlated with rRNA operon copy number (rrN) (23) and extracellular enzyme costs (1) and would decrease more in response to temperature in organisms with simpler metabolisms. To test these hypotheses, we measured the CUE of 23 soil bacterial species representing seven phyla and a 50-fold difference in maximum growth rates on substrates from pyruvate to potato dextrose broth. We then explored correlations between CUE and gene abundances using a comparative genomics approach both to test *a priori* hypotheses and to discover potential markers of CUE using an explore and validate method.

## RESULTS

### Variability in CUE.

The bacterial isolates used in this study were primarily derived from temperate forest soil (Table 1) and were chosen to be representative of the diversity found in the soils they were derived from (35). CUE was determined using optical density measurements of exponentially growing cultures to quantify growth and infrared gas analysis to measure carbon dioxide production rates. Assay conditions included growth at 15, 20, and 25°C on the four substrates glucose, pyruvate, succinate, and potato dextrose broth (PDB).

**TABLE 1:** Isolates used in CUE measurements^*a*^

Isolate	IDTAXA (GTDB) taxonomy	IMG taxon ID	% completeness (% contamination)	Explore or validate	Source or reference
AN5 +	*Bacteria*; *Proteobacteria*; *Alphaproteobacteria*; *Rhizobiales*; *Rhizobiaceae*; *Agrobacterium*	2617270923	99.98 (1.177)	Explore	This study
AN6A +	*Bacteria*; *Proteobacteria*; *Alphaproteobacteria*; *Rhizobiales*; *Rhizobiaceae*; *Agrobacterium*	2619618868	99.96(0.141)	Explore	This study
GAS188 +	*Bacteria*; *Proteobacteria*; *Alphaproteobacteria*; *Rhizobiales*; *Beijerinckiaceae*; EF018539	2693429787	97.806 (2.194)	Explore	88
GAS232 +	*Bacteria*; *Acidobacteriota*; *Acidobacteriae; Acidobacteriales*; *Acidobacteriaceae*; *Terriglobus*	2690315654	100 (3.586)	Explore	This study
EB95 +	*Bacteria*; *Acidobacteria*; *Acidobacteria*; *Acidobacteriales*; *Acidobacteriaceae*; unclassified *Acidobacteriaceae*	2747843220	99.238 (1.724)	Explore	This study
MT12 +	*Bacteria*; *Proteobacteria*; *Alphaproteobacteria*; *Rhizobiales*; *Xanthobacteraceae*; *Bradyrhizobium*	2690316366	99.871 (2.506)	Explore	This study
MT45 +	*Bacteria*; *Actinobacteriota*; *Actinobacteria*; *Corynebacteriales*; *Jatrophihabitantaceae*; MT45	2690315646	95.755 (1.402)	Explore	This study
GAS332 +	*Bacteria*; *Proteobacteria*; *Gammaproteobacteria*; *Betaproteobacteriales*; *Burkholderiaceae*; *Paraburkholderia*	2695420918	99.95 (1.02)	Explore	This study
GAS474 +	*Bacteria*; *Verrucomicrobiota*; *Verrucomicrobiae*; *Methylacidiphilales*; GAS474; GAS474	2690315640	99.324 (4.392)	Explore	89
GAS479 +	*Bacteria*; *Firmicutes*; *Bacilli A*; *Paenibacillales*; *Paenibacillaceae*; *Paenibacillus O*	2693429825	99.511 (0.349)	Explore	This study
GAS525 +	*Bacteria*; *Proteobacteria*; *Alphaproteobacteria*; *Rhizobiales*; *Xanthobacteraceae*; *Bradyrhizobium*	2740892596	99.984 (1.599)	NA	This study
GP183 +	*Bacteria*; *Firmicutes*; *Bacilli A*; *Paenibacillales*; *Paenibacillaceae*; *Paenibacillus E*	2690316367	97.849 (1.613)	Explore	This study
GAS106B+	*Bacteria*; *Proteobacteria*; *Gammaproteobacteria*; *Betaproteobacteriales*; *Burkholderiaceae*; *Paraburkholderia*	2690315676	99.95 (0.827)	Validate	This study
24-YEA-27+	*Bacteria*; *Proteobacteria*; *Alphaproteobacteria*; *Rhodobacterales*; *Rhodobacteraceae*; 24-YEA-8	2767802438	94.838 (1.313)	Validate	This study
BS19 =+	*Bacteria*; *Proteobacteria*; *Gammaproteobacteria*; *Enterobacterales*; *Enterobacteriaceae*; *Ewingella*	2806310493	99.983 (0.536)	Validate	This study
BS40 =+	*Bacteria*; *Actinobacteria*; *Actinobacteriota*; *Actinobacteria*; *Actinomycetales*; *Micrococcaceae*; MA-N2	2806310496	99.039 (1.462)	Validate	This study
BS60 =+	*Bacteria*; *Proteobacteria*; *Alphaproteobacteria*; *Rhizobiales*; *Rhizobiaceae*; P6BS-III	2806310495	100 (0.435)	Validate	This study
BS71 =+	*Bacteria*; *Actinobacteriota*; *Actinobacteria*; *Actinomycetales*; *Microbacteriaceae*; unclassified *Microbacteriaceae*	2806310494	98.99 (0.631)	Validate	This study
*Arthrobacter* *alpinus*	*Bacteria*; *Actinobacteria*; *Actinobacteria*; *Micrococcales*; *Micrococcaceae*; *Arthrobacter*	2634166197	99.541 (1.95)	Validate	90
*Chitinophaga* *pinensis*	*Bacteria*; *Bacteroidetes*; *Sphingobacteriia*; *Sphingobacteriales*; *Chitinophagaceae*; *Chitinophaga*	644736340	99.507 (0.739)	Validate	91
GAS86 +	*Bacteria*; *Proteobacteria*; *Gammaproteobacteria*; *Betaproteobacteriales*; *Burkholderiaceae*; *Paraburkholderia*	2695421038	99.95 (2.108)	Validate	This study
GP187 +	*Bacteria*; *Planctomycetes*; *Planctomycetia*; *Planctomycetales*; *Isosphaeraceae*; *Singulisphaera*	2695420965	99.612 (5.814)	Validate	This study
*Nocardioides* *jensenii*	*Bacteria*; *Actinobacteria*; *Actinobacteria*; *Propionibacteriales*; *Nocardioidaceae*; *Nocardioides*	2731957589	98.698 (1.215)	Validate	92

a Taxonomy is based on 16S sequence assignment using IDTAXA (93). The explore/validate column denotes whether the organism was selected to identify candidate genomic markers in an exploratory approach or appeared only as part of the data set used to determine if those markers were predictive. “NA” indicates that the isolate did not grow on glucose and thus was not used for identifying genomic markers. “+” indicates an isolate from Harvard Forest; “=” indicates that the genome was sequenced using PacBio for this project. ID, identifier.

**FIG 1:**
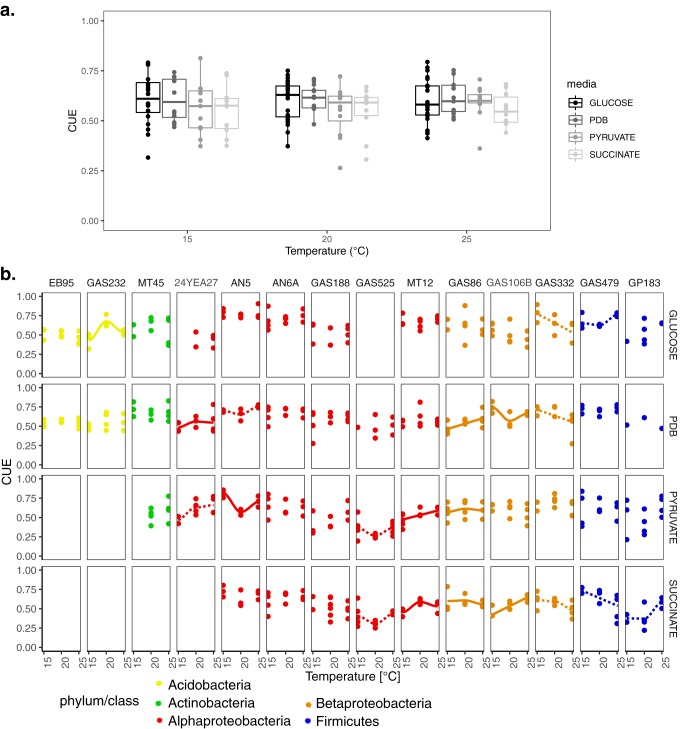
The effect of varying temperature and substrate on the CUE of soil bacteria. (a) Tukey-style (87) box plot of mean CUE for all isolates with at least two experimental replicates, where each point corresponds to one isolate. (b) CUE for individual experimental replicates for each isolate across temperatures; only isolates assayed on substrates other than glucose are shown. Linear and parabolic curves were *t* and compared for each scenario, and curves were assigned if slope parameters were statistcially significant. ANOVA was used for comparisons between models to determine if the more complex parabolic model *t* was significantly better than the simpler linear one. Loess-smoothing curves representing a significant parabolic *t* (dashed, *P* < 0.1; solid, *P* < 0.05) or lines depicting a significant linear *t* across the three temperatures are overlaid. Solid line, *P* < 0.05; dashed line, *P* < 0.1.

CUE varied from 26% to 81% across conditions (Fig. 1a). This variation in CUE was underlain by substantial variation among taxa in both respiration and growth rates, which did not always correlate with one-another (see Fig. S1 in the supplemental material). Although our level of replication was relatively low, we did not find evidence that CUE exhibited a multimodal distribution under any of the assay conditions using a Hartigan’s dip test (36), except for PDB at 15°C (Fig. S2). CUE showed a weak positive correlation with growth rate during CUE measurements on glucose (repeated measures correlation *r* = 0.26, *P* < 0.001, 270 df), PDB (*r* = 0.22, *P* = 0.02, 120 df), pyruvate (*r* = 0.24, *P* < 0.01, 114 df), and succinate (*r* = 0.25, *P* < 0.05, 92 df). CUE was more strongly negatively correlated with mass-specific respiration rate, with correlation coefficients between −0.41 (PDB; *P* < 0.001, df = 119) and −0.58 (glucose; *P* < 0.001; df = 206) across the substrates tested. Thus, variation in respiration among taxa was more strongly correlated with variation in CUE among taxa than was growth rate.

10.1128/mBio.02293-19.1 FIG S1 Responses of mass-specific respiration and growth rates to changes in substrate and temperature. Lines are colored by isolate such that each point represents the mean respiration rate and growth rate for a given temperature and substrate for an isolate. Lines are drawn to connect points corresponding to a given isolate and do not imply a statistical relationship. Download FIG S1, PDF file, 0.1 MB. Copyright © 2020 Pold et al. 2020 Pold et al. This content is distributed under the terms of the Creative Commons Attribution 4.0 International license.

10.1128/mBio.02293-19.2 FIG S2 Frequency histograms of CUE of isolates grown on the four substrates at three temperatures. Each count represents the average of results from all replicates for a given isolate under that assay condition. Download FIG S2, PDF file, 0.05 MB. Copyright © 2020 Pold et al. 2020 Pold et al. This content is distributed under the terms of the Creative Commons Attribution 4.0 International license.

### Effect of substrate quality on CUE and its temperature sensitivity.

CUE did not increase significantly with the energy content of the substrate (heat of combustion standardized to C content; Fig. S3), likely because isolates differed in their ability to grow on the different substrates (Fig. S1). The effects of temperature and substrate on CUE were isolate specific [temperature * isolate interaction F(22,476) = 2.324, *P* < 0.001; substrate * isolate interaction F(32,476) = 2.100, *P* < 0.001 for three-way analysis of variance (ANOVA); Fig. 1b]. This effect was underlain by variation in both the growth rate and respiration rate of the isolates (Fig. S1) with temperature.

10.1128/mBio.02293-19.3 FIG S3 Effect of C quality on CUE. Each line denotes values for a different isolate. The *x* axis data represent the heat of combustion of the substrate in kilojoules per mole divided by the number of C atoms in a mole of the substrate. Only cultures grown at 20°C are plotted. Download FIG S3, PDF file, 0.1 MB. Copyright © 2020 Pold et al. 2020 Pold et al. This content is distributed under the terms of the Creative Commons Attribution 4.0 International license.

**FIG 2:**
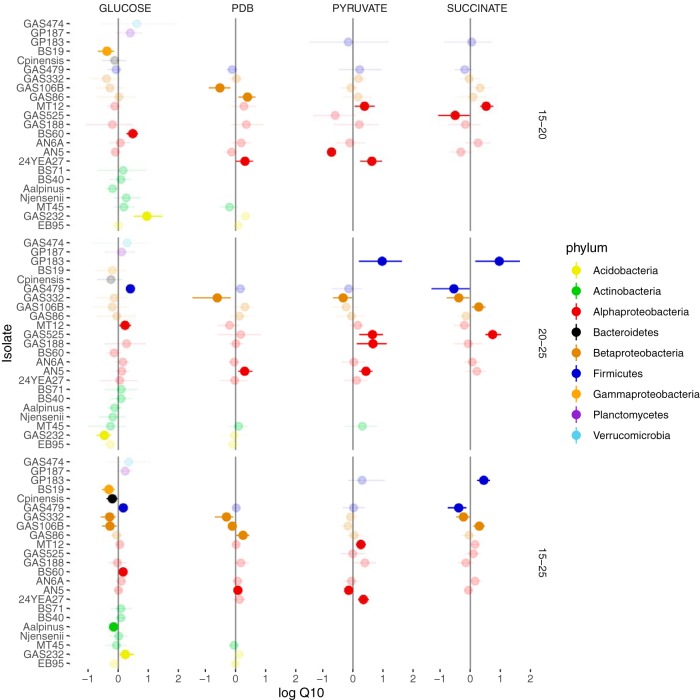
*Q*_10_ of CUE across three temperature ranges assayed in this study. Values are presented as the logarithm in order to center them on zero and are colored according to substrate. The intensity of the color is halved where the 95% bootstrapped confidence intervals on the estimate of the raw data overlap 1 (i.e., where CUE is insensitive to temperature), depicted here as a horizontal line.

Across all isolates and substrates, the *Q*_10_ value of CUE (i.e., the factor by which soil respiration increased by a 10°C increase in temperature) ranged from 0.49 to 2.63 (Fig. 2), equivalent to a halving to a more than doubling in its value. In 71% of the cases, however, CUE was unaffected by temperature over the range studied, indicating that respiration and growth often responded similarly to temperature. CUE did not differ with respect to mean temperature sensitivity for the different substrates (Fig. S4) or with respect to the frequency with which the 95% bootstrap confidence intervals did not overlap 1 for a given isolate by temperature combination (Fig. 2).

10.1128/mBio.02293-19.4 FIG S4 Phylogenetically weighted mean temperature sensitivity of CUE for the four substrates and three temperature ranges used in this study, reported with 95% confidence intervals. Reported means and confidence intervals represent the posterior estimates resulting from running an animal model in MCMCglmm. Download FIG S4, PDF file, 0.1 MB. Copyright © 2020 Pold et al. 2020 Pold et al. This content is distributed under the terms of the Creative Commons Attribution 4.0 International license.

### Phylogenetic conservatism of CUE and its temperature sensitivity.

A maximum likelihood-based phylogenetic tree was built for the isolates based on a set of conserved single-copy genes (37) using RAxML (38). This tree was used as the backbone for identifying whether the CUE values observed were distributed at random in the bacterial taxa studied (Pagel’s lambda = 0), whether the values were consistent with evolution following Brownian motion (Blomberg’s *K* = 1, Pagel’s lambda = 1), or whether they were comparably underdispersed (Blomberg’s *K* > 1) or overdispersed (*K* < 1) on the phylogenetic tree. CUE did not have a consistent phylogenetic signal—it varied with temperature and substrate. Pagel’s lambda differed from zero only at 25°C and not at 15 or 20°C on glucose, although the confidence intervals were large (Table 2). On pyruvate, however, CUE correlated with phylogeny and approached the expected level under conditions of Brownian motion based on Pagel’s lambda for all temperatures. Blomberg’s *K* was typically small and less frequently different from zero than would be expected by chance, indicating that variation in CUE cannot be decisively said to vary more within than between clades in our data set. Reflecting this generally weak phylogenetic signal, knowing the CUE of all remaining taxa did not help predict CUE in adjacent tips on the phylogeny except for taxa grown on glucose or pyruvate at 15°C or for the *Q*_10_ between 20 and 25°C for taxa grown on glucose (Fig. S5). The estimation error for CUE on glucose was not correlated with distance to the nearest sampled taxon, although it was positively correlated for pyruvate at 15°C (ρ = 0.85, *P* < 0.01). The estimation error for *Q*_10_ CUE on glucose was weakly positively correlated with phylogenetic distance (ρ = 0.37, *P* < 0.1) but was more strongly negatively correlated for pyruvate between 15 and 20°C (ρ = 0.67, *P* < 0.05).

**TABLE 2:** Phylogenetic signal of CUE and its temperature sensitivity over a range of temperatures and substrate types^*a*^

Substrate	Temp (^o^C)	K (p)	λ (p)	Range (^o^C)	K (p)	λ (p)
	15	0.11	0.4	15–20	0.25	0.33
glucose	20	0.11	0.8	20–25	0.70*	0.98***
	25	0.21	0.86**	15–25	0.2	0.52
	15	0.1	0.48•	15–20	0.05	0.19
PDB	20	0.83	0.88**	20–25	0.01	0
	25	0.11	0.65*	15–25	0.02	0
	15	0.66**	0.99**	15–20	0.15	0.99**
pyruvate	20	0.31•	0.98**	20–25	0.22	0.81
	25	0.38•	0.99***	15–25	0.19	0.99**
	15	0.17	0.62•	15–20	0.04	0.99**
succinate	20	0.28•	1.00**	20–25	0.11	0.97*
	25	0.1	0.89*	15–25	0.03	0

a “Temperature” denotes CUE at that temperature, while “range” denotes how CUE changed over the temperature range denoted. “K” denotes Blomberg’s K, while λ denotes Pagel’s lambda. Values for which the *P* value for a test comparing values to zero is greater than 0.05 are in gray, while the asterisks that follow values in black denote *P* < 0.05 (*), *P* < 0.01 (**), or *P* < 0.001 (***). Bullets (•) indicate *P* < 0.1. The 95% confidence intervals of K are 0.36 to 2.46, 0.32 to 2.45, 0.26 to 2.49, and 0.19 to 2.49 for a Brownian process simulated on the glucose, PDB, pyruvate, and succinate trees, respectively. The corresponding values for lambda are 0.89 to 1, 0.89 to 1, 0.9 to 1, and 0.8 to 1.

10.1128/mBio.02293-19.5 FIG S5 Plot of observed mean CUE (A) or CUE *Q*_10_ (B) values for each isolate and incubation condition versus the predicted mean CUE based on phylogenetic reconstruction using ancestral reconstruction techniques. Each point represents an isolate, the *x* axis the observed mean CUE, and the *y* axis the mean CUE predicted for the isolate based on ancestral reconstruction. The 1:1 line, indicating perfect agreement between the predicted and observed CUE data, is drawn in solid gray, and the correlation for significant relationships between observed and predicted mean CUE for each isolate is drawn as a heavier line. Spearman’s rho is presented for those correlations. ***, *P* < 0.001; **, *P* < 0.01; *, *P* < 0.05; •, *P* < 0.1. Download FIG S5, PDF file, 0.1 MB. Copyright © 2020 Pold et al. 2020 Pold et al. This content is distributed under the terms of the Creative Commons Attribution 4.0 International license.

### Drivers of CUE.

**FIG 3:**
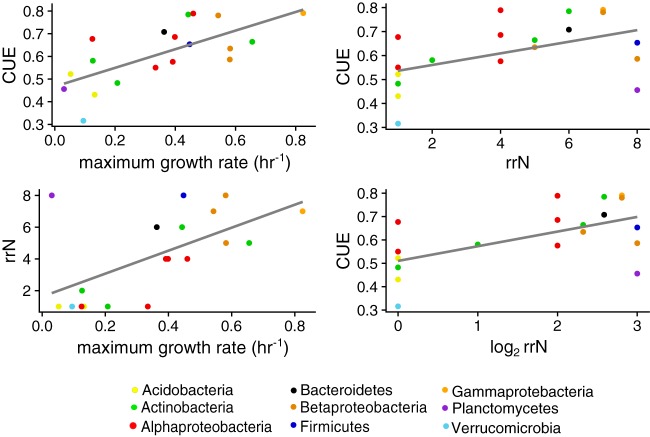
Relationships between CUE, growth rate, and rrN for bacteria grown on glucose at 15°C. Full details can be found in Table 3.

We annotated the bacterial genomes using IMG (39) and then tested the *a priori* hypotheses that CUE would be negatively correlated with rrN, growth rate, and extracellular enzyme investment but positively correlated with metabolic complexity. Maximum observed growth rate (0.01 to 0.56 h^−1^), rrN (1 to 8 copies), and CUE were found to be frequently positively correlated with one another on glucose (Table 3) (Fig. 3; see also Fig. S6). CUE was not correlated with extracellular enzyme activity (EEA) or with extracellular gene or transporter gene density (*P* > 0.2). Likewise, we did not find a correlation between *in silico* estimated CUE for extracellular enzyme production—which we determined using amino acid biosynthesis and polymerization costs (see Materials and Methods)—and the CUE observed on glucose, except at 15°C (Table 3). Codon bias is a measure of the degree to which a genome is optimized for rapid and efficient translation (40); based on the growth rate-yield hypothesis, codon bias is expected to correlate negatively with CUE. However, the observed relationship between codon bias and CUE paralleled the nonnegative correlation observed between CUE and maximum growth rate (Fig. S7). We found evidence for a positive correlation between CUE and the number of metabolic pathways (32) on glucose (0.002 CUE metabolic pathway^−1^; *P* < 0.001) at 15°C (Table 3) and for a weaker nonsignificant positive correlation at 25°C (0.001 CUE metabolic pathway^−1^, *P* < 0.1). The overall functional gene composition that an organism had was correlated with CUE on glucose at both 15 and 25°C, as evidenced by a significant correlation between the nonmetric multidimensional scaling (NMDS) coordinates of a taxon’s genome size-standardized KEGG orthology (KO) composition and its CUE (*R*^2^ = 0.43, 0.29, *P* < 0.05; envfit in vegan).

**TABLE 3:** Regression coefficients for a phylogenetic generalized least-squares model fit to CUE on glucose at a given temperature versus rrN or the maximum growth rate observed across all assay conditions^*a*^

Temp (°C)	CUE vs GR_max_	CUE vs rrN	rrN vs GR_max_	CUE vs log_2_ rrN	Metabolic pathway count	CUE for EEA production
15	0.41***	0.028*	7.23**	0.071**	0.0022***	2.782*
20	—	—	6.18*	0.039**	—	—
25	0.26*	0.021*	6.18*	0.052**	0.001.	—

a Slopes are shown for the cases in which the *P* value was less than 0.1 (•), 0.05 (*), or 0.01 (**); dashes (—) indicate that the slope was not statistically significant. Metabolic pathway count data correspond to the number of MAPLE pathways with 80% completeness. CUE for EEA production corresponds to the theoretical fraction of C from glucose expected to be retained in the extracellular enzymes produced by the organism rather than being burned to produce the ATP needed to make the corresponding amino acids *de novo* and then polymerize them into the proteins. GR_max_, maximum growth rate.

10.1128/mBio.02293-19.6 FIG S6 Correlation between CUE and maximum growth rate of taxa across the four substrates and three temperatures assayed. PGLS slopes are drawn, with numbers on each panel denoting the slope and its significance (***, *P* < 0.001; **, *P* < 0.01; *, *P* < 0.05; •, *P* < 0.1). Download FIG S6, PDF file, 0.2 MB. Copyright © 2020 Pold et al. 2020 Pold et al. This content is distributed under the terms of the Creative Commons Attribution 4.0 International license.

10.1128/mBio.02293-19.7 FIG S7 Correlations between CUE and codon bias (determined as described previously by Vieira-Silva and Rocha [39]) for bacterial isolates grown on glucose. Spearman correlation coefficients (not corrected for phylogenetic correlation) are included, along with the *P* value (***, *P* < 0.001; **, *P* < 0.01; *, *P* < 0.05; •, *P* < 0.1). Download FIG S7, PDF file, 0.2 MB. Copyright © 2020 Pold et al. 2020 Pold et al. This content is distributed under the terms of the Creative Commons Attribution 4.0 International license.

We also completed an “exploratory” analysis for markers of CUE, where we looked at all KO categories and maps to see whether they were correlated with CUE for a subset of isolates grown on glucose, and then validated them based on their consistent appearance in additional isolates and under alternative cultivation conditions. These cultivation conditions included both the growth of bacterial isolates on the remaining substrates and the growth of soil-derived mixed bacterial communities in microcosms of artificial soil, where CUE was measured using the ^18^O-H_2_O method (41; also L. A. Domeignoz-Horta, G. Pold, X. L. Liu, S. D. Frey, J. M. Melillo, K. M. DeAngelis, submitted for publication). No markers proposed by our exploratory analysis were uniformly validated by these methods (Fig. S8).

10.1128/mBio.02293-19.8 FIG S8 Venn diagrams of numbers of individual KO markers (A) or pathways (B) whose genomic density is positively or negatively correlated with CUE. In all instances, “glucose explore” is considered to consist of the proposed markers of efficiency whereas the remaining three data sets are considered to be “validating” data sets. Sample sizes (in numbers of isolates or microcosms) for each analysis were as follows: for “other” substrates, 10 to 13 samples; for “glucose explore,” 13 samples; for “glucose all,” 22 samples; for microcosms, 10 samples. Download FIG S8, PDF file, 0.4 MB. Copyright © 2020 Pold et al. 2020 Pold et al. This content is distributed under the terms of the Creative Commons Attribution 4.0 International license.

### Drivers of *Q*_10_.

**FIG 4:**
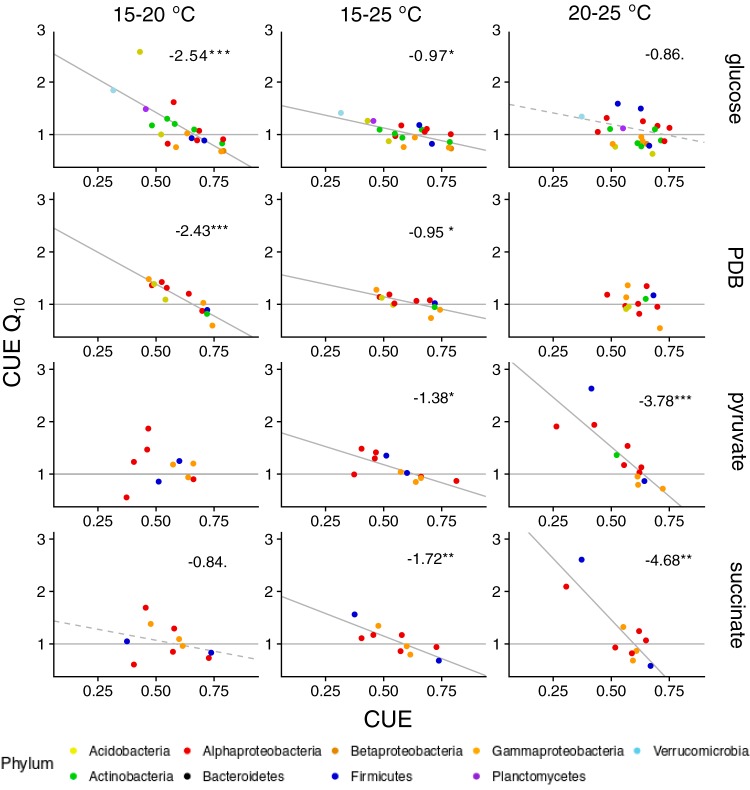
Change in CUE with temperature compared to the CUE at the starting temperature (i.e., *Q*_10_ between 15 and 20°C or 15 to 25°C against CUE at 15°C or *Q*_10_ between 20 and 25°C compared to CUE at 20°C). The coloring of the points is based on phylum (or on class for *Proteobacteria*), with each point representing the mean CUE and *Q*_10_ for each isolate under the relevant treatment and temperature conditions. Solid gray lines represent the nonphylogenetic linear model and dashed black lines the phylogenetic generalized least-squares *t*. Numbers on each plot denote the slope of the phylogenetic regression for *P* values below 0.1 (***, *P* < 0.001; **, *P* < 0.01; *, *P* < 0.05; •, *P* < 0.1). The thin gray horizontal line denotes no temperature sensitivity (i.e., *Q*_10_ = 1), so points above the line indicate an increase in CUE with temperature, and points below the line a decrease.

The *Q*_10_ of CUE tended to be lower for more efficient taxa (Fig. 4). This led to a homogenization of CUE at higher temperatures, with the standard error of CUE comparisons between isolates decreasing between 15 and 20°C and 15 to 25°C for all substrates. The temperature sensitivity of CUE on glucose was negatively correlated with the number of metabolic pathways at 15 to 20°C (0.007 decrease in *Q*_10_ for every additional pathway, *P* < 0.01) and at 15 to 25°C (0.002 decrease, *P* < 0.05) but not at 20 to 25°C. This corresponds to an expected decrease in CUE of 14% between 15 and 20°C for the isolate with the greatest number of metabolic pathways (*Ewingella* BS19; *n* = 200) and an increase of 6% for the bacteria with the fewest (*Verrucomicrobium* GAS474; *n* = 49). Extracellular enzyme-related functions increased the temperature sensitivity of CUE only for the 15 to 20°C temperature range on glucose (*Q*_10_ increases 0.01; extracellular enzyme Mbp^−1^, *P* < 0.01). *Q*_10_ of CUE was not consistently correlated with genomic density of transporters (15 to 20°C, −0.016 transporters Mbp^−1^; 20 to 25°C, +0.008 transporters Mbp^−1^) and did not correlate with maximum growth rate or log_2_rrN (see Table S1 in the supplemental material).

10.1128/mBio.02293-19.9 TABLE S1 Regression coefficients for a phylogenetic generalized least squares model fit to CUE for a given substrate and temperature range versus ribosomal RNA operon copy number or the maximum growth rate observed across all assay conditions. Slopes are shown for the cases in which the *P* value is less than 0.1 (•), 0.05 (*), or 0.01 (**). Metabolic pathway count data correspond to the number of MAPLE pathways with >80% completeness. CUE for EEA production corresponds to the theoretical fraction of C from glucose expected to be retained in the extracellular enzymes produced by the organism rather than being burned to produce the ATP needed to make the corresponding amino acids *de novo* and then to polymerize them into the proteins. Download Table S1, PDF file, 0.1 MB. Copyright © 2020 Pold et al. 2020 Pold et al. This content is distributed under the terms of the Creative Commons Attribution 4.0 International license.

Our exploratory analysis found the density of genes such as malate synthase (K01638) to be negatively correlated with *Q*_10_ (Table S2), while the set of genes positively correlated with the *Q*_10_ of CUE contained a number of genes encoding transporters. Increased densities of genes involved in N-glycan biosynthesis—or decreased densities of genes associated with alpha-linolenic acid metabolism—were associated with more-positive *Q*_10_ values for CUE between 15 and 20 or 25°C. As for CUE at a fixed temperature, no candidate markers of the temperature sensitivity of CUE were validated by both the microcosms and “other substrates” data sets for either individual KO’s or whole KEGG pathways.

10.1128/mBio.02293-19.10 TABLE S2 Genomic markers of CUE or CUE temperature sensitivity (*Q*_10_) identified in exploratory and complete glucose data sets and confirmed by their presence as correlates of efficiency in the microcosm data set or for at least two of the three remaining substrates in the liquid culture assays. Markers are included for the cases in which the slopes were either both positive or both negative for the two data sets at an alpha of 0.05. Download Table S2, PDF file, 0.1 MB. Copyright © 2020 Pold et al. 2020 Pold et al. This content is distributed under the terms of the Creative Commons Attribution 4.0 International license.

## DISCUSSION

We hypothesized that CUE would be highly variable across soil bacteria and temperatures, and this was indeed the case. The range of CUE observed for our soil bacteria was similar to that of fungi isolated from the same research site (E. W. Morrison, unpublished), as well as to that of bacteria isolated from a wide range of ecosystems and domestication histories (23). Nonetheless, the upper limit of efficiency reported for the bacterial taxa that we studied was 10% lower than that for the fungi and almost 30% higher than that for the aforementioned bacteria grown under similar conditions. Furthermore, the range of *Q*_10_ values for the bacteria in the present study included a pair of stronger, more positive temperature responses (*Q*_10_ values of >1) than those reported for fungi, consistent with a previous study which showed bacterial growth to be less negatively affected by higher temperatures than fungal growth (42).

The diverse CUE temperature responses that we identified in this study contrast with the homogeneous response typically assumed in models (3, 43). Although we are not among the first to observe an increase in CUE with temperature for soil microbial taxa (4), the magnitude and range of temperature responses seen across taxa here were up to five times larger than those previously reported for soil communities (4, 8, 44, 45). We suggest that the mute temperature responses observed for mixed communities represent the result of a statistical averaging effect wherein mixtures of substrates (4) and microbial taxa (6, 7) with divergent CUE cancel each other out. This is consistent with the observation that CUE on phenol (which can be used by a more restricted group of taxa [46, 47]) showed a much stronger response to temperature than CUE on the more ubiquitously used glucose (6). Consequently, taxon-level differences in CUE temperature sensitivity may matter for bulk soil organic matter cycling only in cases in which they are linked to the presence of other “response” traits which narrow the diversity of the active community. For instance, bacterial responses to drought and nitrogen (N) addition are more similar than expected in closely related taxa (48).

As a complex physiological trait which integrates the entire metabolic network of a cell, we hypothesized that CUE and its temperature sensitivity would be more similar in closely related organisms than expected by chance. This pattern was observed, but the degree of phylogenetic conservation was insufficient for building a predictive model of CUE in unsampled tips of the phylogeny. The poor ability to predict CUE based on phylogeny contrasts with other complex physiological traits such as oxygenic photosynthesis (34) and with apparent growth-limiting traits such as rrN (23, 49), which are assumed to be phylogenetically conserved because of low horizontal gene transfer frequency. While some of the uncertainty in predicted CUE values can be attributed to undersampling of the phylogenetic tree (50), this is unlikely to be the sole factor because estimation errors were never strongly positively correlated—and were sometimes negatively correlated—with distance to the nearest sampled taxon on the tree. Independently of the reason, the pattern of CUE on the tree is inadequately modeled by Brownian motion using the scale of data available here. We proceeded to explore whether CUE varies as a function of additional genomic traits.

The high-efficiency and low-efficiency organisms had different overall metabolic potentials, but few of the specific traits that we had proposed *a priori* to be correlated with CUE were actually correlated in the manner hypothesized. Of particular note is rrN, which has received considerable attention for its apparent role in setting the upper limit on growth rate (25, 26, 40) and, in turn, in determining the ecological strategy and bacterial CUE under conditions of high nutrient availability (23). Although we observed the expected positive correlation between rrN and maximum growth rate or codon bias, our results diverge from those reported from previous studies (23, 32) in that CUE was positively correlated with rrN and maximum growth rate under many temperature-substrate combinations. The negative correlation between growth rate and CUE under conditions of high resource availability was initially proposed based on the better-studied and oft-observed growth rate-yield trade-off (23, 24) whereby faster-growing organisms produce less biomass for a given amount of substrate. This growth rate-yield trade-off is thought to be the consequence of balancing the speed and accuracy of translation (23, 26) and has been proposed to be a central component of the copiotroph-oligotroph niche axis (51). Nonetheless, we are not the first to question (52) the ubiquity of the growth rate-yield trade-off.

Additional work has shown that bacteria can attain rapid growth under conditions of both high-efficiency and low-efficiency metabolisms. For example, selection for rapid growth in Escherichia coli can result in either a high-uptake, low-yield phenotype or a moderate-uptake, high-yield phenotype (31). Rather, the growth rate-yield trade-off does not act alone to determine efficiency and instead acts in concert with a second axis describing the relationship between substrate uptake rate and yield (53). As a result, the ubiquity of overflow metabolism—oft cited as a cause for the rate yield trade-off—is uncertain, as not all organisms shift away from the pentose phosphate pathway and toward glycolysis when grown on glucose (53). Since we found a similar pattern of CUE increasing with growth rate and rrN even for nonfermentable substrates, this indicates that overflow metabolism was not a uniform driver of CUE and yield at high growth rates in our environmental isolates. This is also consistent with other studies which used aquatic isolates: maximum growth rate and yield were negatively correlated only for a subset of *Proteobacteria* in one study (32) and were positively correlated for *Bacillus* species in another (52). Mechanistically, it would make sense that CUE increases with growth rate if (time-dependent) maintenance respiration outstripped (time-independent) growth respiration (13), but neither the literature (54) nor the values derived from the current data set indicate this to be the case. Furthermore, maintenance respiration rates would have to be lower in fast-growing taxa than in slow-growing taxa to explain the higher CUE in fast-growing taxa, which contradicts the pattern previously observed by Van Bodegom (55). Therefore, the mechanisms underlying the positive correlation between growth rate and efficiency in the present isolates remain unclear and are unlikely to be the same as those proposed for growth rate versus yield.

Our exploratory analysis searching for genomic markers of efficiency also failed to provide a substantive explanation for the observed differences in CUE between taxa. In contrast to other studies (23, 32), we generated data to validate the genomic correlates of CUE proposed by an initial data exploration. None of the markers proposed on the basis of their correlation with CUE on glucose were consistently validated by the other data sets, and those which were validated by one or the other were often found in few genomes and/or formed isolated steps in metabolism. This lack of validation by additional data sets may in part be a consequence of that fact that the substrates differ in the point at which they enter central metabolism, just like the various substrates found in soils. Glucose may enter any of a number of pathways with different N requirements, energy yields (21), and anabolic potentials—including through the tricarboxylic acid (TCA) cycle where pyruvate and succinate are generated—whereas pyruvate and succinate are much more limited in the diversity of pathways that they can directly enter. On the other hand, the presence of interspecific interactions may explain why glucose-fed isolates and cellobiose-fed microcosms differed in the genes correlated with CUE, despite the substrates being able to enter the same metabolic pathways (56). For instance, genes whose presence is advantageous for growth in isolation may not be beneficial in a mixed community (57, 58) or where substrates are not in a freely available pool (59, 60). Furthermore, while we are certain that CUE was measured during the exponential phase for the isolates, such was unlikely to be the case for the soil communities, which were left without substrate addition for a month prior to CUE measurements. Finally, it is possible that the metagenomes inferred for the microcosm communities based on their 16S rRNA gene content do not adequately represent the true metagenomic content, as even very similar 16S genes can be associated with different functional compositions (61). Nonetheless, our results indicate that the taxa which are most efficient on one substrate are unlikely to be the most efficient on another, such that CUE is more like a dynamic response variable than a fixed ecological trait. Given that CUE varied substantially as a function of substrate—and that substrate chemistry can differ substantially across soils (62)—it is possible that the temperature sensitivity of CUE, rather than its absolute value, is more useful for comparing the physiologies of various microbial communities that differ in composition.

The temperature sensitivity of CUE was not consistently correlated with common soil-associated traits such as extracellular enzyme gene allocation but could be predicted based on the value of CUE itself. Specifically, the temperature sensitivity of CUE was negatively correlated with basal CUE under many assay conditions. This could not be attributed to differences in the growth rates of organisms at the lower temperature, indicating that increasing temperatures do not preferentially favor slow-growing taxa. Decreased CUE temperature sensitivity with greater basal CUE does, however, indicate that the CUE of communities should homogenize at higher temperatures, as inefficient communities show increases in efficiency and efficient ones show decreases. Nonetheless, given the possibility of different substrates becoming available at higher temperatures and the substrate-specific divergences in CUE across taxa, the correlation between CUE and its temperature sensitivity is unlikely to hold for intact microbial communities. Accordingly, Zheng et al. (4) found only a very weak negative correlation between CUE and its *Q*_10_ values across a range of different soils. Future work integrating the diversity of temperature responses to predict the outcome of community interactions is necessary to advance the field.

### Conclusion.

We found that fast-growing taxa are likely to grow more efficiently and that highly efficient taxa tend to decrease in efficiency with temperature more than those with initially low CUE. Therefore, our results are consistent with the hypothesis that maintenance respiration is a more pivotal factor in regulating soil bacterial CUE than previously recognized. Our results also challenge the idea that high ribosomal operon copy number correlates with reduced growth efficiency. Previously and *de novo* hypothesized markers of efficiency were not consistent across assay conditions, reinforcing the hypothesis that CUE represents an integrator of bacterial physiology in response to the environment rather than a fixed descriptor of their niche and that some bacteria are not intrinsically more efficient than others. Our results also suggest that the communities capable of effectively retaining soil C at a given time point might not necessarily be the best equipped to continue to do so in the future, because the taxa able to grow most efficiently at low temperature tended to release more substrate as CO_2_ as the incubation temperature increased. Our report therefore opens the door for additional work with isolates under the more realistic soil conditions that we ultimately wish to understand.

## MATERIALS AND METHODS

### Isolate selection.

We used a total of 23 bacterial isolates from our laboratory culture collection and from public culture collections for our study (Table 1). The 20 isolates from our laboratory collection were derived from the organic and A-horizon of the Canton series underlying a temperate deciduous forest stand at the Harvard Forest Long-Term Ecological Research (LTER) site, in Petersham, MA. These bacteria were isolated under a range of cultivation conditions (63), and freezer stocks were prepared using the second or third streak of the original soil-derived colony. The isolates used were selected to cover the global diversity of soil bacteria (64). The genomes were sequenced, and a phylogenetic tree was built following the methods described in the supplemental material.

### CUE measurement.

To measure bacterial CUE, isolates were grown in triplicate on up to four substrate types at a pH of 6; this is the lowest pH at which all isolates were able to grow but was still 2 pH units higher than that of the soil from which most of them had originally been isolated. The substrate types were potato dextrose broth (PDB) and glucose, pyruvate, and succinate media. We ensured that the cells were acclimated by transferring exponentially growing cultures by the use of the temperature and media used for assay conditions at least three times prior to taking CUE measurements. Additional information on media and assay setup can be found in the supplemental material.

The optical density and respiration rate of cultures were monitored throughout the exponential-growth phase using a Spectronic-20 spectrophotometer at 600 nm and a Quantek Instruments model 906 CO_2_ analyzer, respectively. Prior to each read, tubes were subjected to vigorous vortex mixing to ensure that the solution and headspace CO_2_ were in equilibrium. At least three distinct experiments starting with a new freezer stock restreak were completed for each isolate and condition assayed. A conversion factor of 130 g C optical density (OD)^−1^ ml^−1^ was used to calculate microbial biomass carbon (MBC) throughout the growth curve (BioNumber 109836), as technical challenges encountered in collecting biomass from cultures meant that MBC was underestimated in the taxa characterized by small cells. Therefore, we decided to use one nonideal biomass conversion factor rather than 23 of them.

Calculation of CUE was restricted to the exponential-growth phase, which was identified by taking the natural logarithm of biomass versus time and finding the range of time points which maximized the slope. Three to ten time points were used per curve for this purpose, depending on the growth rate of the isolate and the duration of the exponential-growth phase. When the slope of the growth rate or mass-specific respiration rate did not differ from zero (F test *P* > 0.05), the data were discarded and the experiment was repeated. Only those assay conditions for which at least two replicates satisfied these criteria were included in our analysis. CUE was calculated as CUE = μ/(μ + *R*), where μ is the intrinsic rate of increase, calculated as the slope of ln(biomass) against time, and *R* is the mass-specific respiration rate during the same time period (Morrison et al., submitted). This is similar to the method used previously by Keiblinger et al. (65); the use here of multiple CO_2_ measurements and of the entire exponential-growth phase is expected to have improved estimate reliability. We used repeated-measures correlations (66) to look at the effect of substrate quality.

### Data analysis.

**(i) Calculating temperature sensitivity.** Although our experimental design was such that the same starting culture would be incubated in four different substrates under three different temperatures, successful concurrent cultivation under all 12 conditions was rarely achieved. Therefore, in the absence of such a blocked design, bootstrapping was used to determine uncertainty in the temperature sensitivity of CUE. In other words, the temperature sensitivity of CUE was calculated for all combinations of 15 and 25°C for a given substrate and isolate combination, and the standard error was calculated from this.

**(ii) Genome annotation.** Genome annotation was completed using the Joint Genome Institute’s IMG pipeline (39). Open reading frames (ORFs) potentially encoding extracellular enzymes were identified based on the presence of signal peptides using SignalP 4.1 (67) with the default *d* cutoff value of 0.57. This subset of ORFs was then examined for enzymes involved in litter and necromass decomposition. Carbohydrate-active and lignin-degrading enzymes were identified using dbCAN (68) v6, and additional putative extracellular enzymes were extracted by name from the IMG annotations using “*rotease” or “*roteinase” or “*eptidase” or “*osphatase” or “*hospholipase” annotation as the keyword string(s). These extracellular enzyme classes were chosen to retain consistency with functions typically assayed in soils.

Transporters were annotated using TCDB (69) and TransportDB 2.0 (70) and summed for each isolate. Identification was completed with gBlast2 (71) against the TCDB reference database downloaded on 20 July 2018 and using the TransAAP online tool against TransportDB in August of 2018. The number of metabolic pathways possessed by an organism as assessed on the basis of annotations with MAPLE (72) was used as a proxy for metabolic complexity (32). The envfit function in vegan (73) was used to evaluate whether CUE was correlated with differences in overall KO composition of bacterial genomes, where the initial ordination of functional gene composition was completed using NMDS of Bray-Curtis distances.

**(iii) Protein production costs.** We calculated the total extracellular enzyme cost as a function of amino acid biosynthesis and translation, using the amino acid biosynthesis costs presented previously by Kaleta et al. for E. coli with glucose as the substrate and assuming 4.2 ATP consumed per peptide bond formed (74). Assuming that 26 ATP are produced per six glucose C, we calculated the theoretical C assimilation efficiency for each protein as the ratio of C in the protein to the C in the protein plus CO_2_ respired in the process of making the ATP required to make the protein. The “per protein CUE” for each protein was then weighted by its expected relative expression level to get a whole-exoenzyme production cost. Relative expression levels were predicted on the basis of codon usage bias as outlined in the supplemental material.

### Mixed bacterial communities.

Cells were extracted using soil from the same Harvard Forest LTER site as the bacterial isolates by the use of 224 mM sodium pyrophosphate (75) and were subsequently passed through a 0.8-μm-pore-size mixed cellulose ester syringe filter to remove eukaryotic cells. The filtered cell suspension was then used to inoculate an artificial soil matrix consisting of 70% acid-washed sand, 20% muffled and acid-washed silt, and 10% calcium chloride-treated bentonite clay, initially amended with mixed deciduous leaf litter dissolved organic carbon (DOC), 2× VL55 media (75), VL55 minerals, and yeast extract. The communities were kept at 60% water holding capacity at 15 or 25°C for 4 months, with weekly additions of 0.5 mg g soil^−1^ cellobiose and 0.05 mg g soil^−1^ ammonium nitrate solutions as sources of C and N, respectively, for the first 3 months.

We measured CUE using the ^18^O-water method (41) at the same temperature as that used for the long-term incubations. The bacterial communities were sequenced at the Environmental Sample Preparation and Sequencing Facility at Argonne National Laboratory following the Earth Microbiome Project protocol (76). Metagenomes of these communities were inferred using PICRUSt v 1.1.1 (77), with closed-reference OTUs picked in Qiime v1.9.0 (78) at 99% identity using uclust (79) against Greengenes v. 13.5 (80). We used the relative abundances of the predicted KEGG ortholog gene categories as predictors of CUE. The nearest sequenced taxon index (NSTI) values for the genomes used in the functional assignments averaged 0.02 (range, 0.003 to 0.072).

### Identification of genomic markers.

We focused on identifying markers of CUE on glucose, as this is the substrate on which we were able to get the greatest number bacteria to successfully grow. Genomic markers of efficiency (and temperature sensitivity of efficiency) of glucose utilization were identified and validated in one of two ways. When we had an *a priori* hypothesis about the marker based on the literature, we used the full set of bacteria grown on glucose for our analysis. This was the case for rrN, codon bias (a proxy for growth rate [40]), extracellular enzyme costs, and number of metabolic pathways (32). For the other hypotheses, we used a two-part process: (i) a preliminary exploratory analysis for bacteria grown on glucose to identify candidate markers and (ii) a distinct validation step in which a “validating” data set of bacteria grown on other substrates and a data set of bacterial communities grown in artificial soil on cellobiose were interrogated for the same patterns. This exploratory analysis focused on the 5,270 KEGG orthologues found in our bacterial genomes.

The process of identification and validation of markers in bacterial isolates was completed using phylogenetic generalized least squares in caper v1.0.1 (81). caper uses a maximum likelihood method to infer the branch length transformations of the phylogenetic tree which minimizes phylogenetic correlations of the model residuals, thereby flexibly accounting for different degrees of phylogenetic signal in the residuals of comparable models. Genes were said to be candidate markers of efficiency at an alpha of 0.05 for the slope estimate. We used an identical approach to identify markers of efficiency in our first validating data set, which consisted of genes similarly correlated with CUE in at least two of the three other substrates. This criterion was selected to balance ensuring the robustness of the markers over multiple substrates with the fact that different substrates are likely to enter different metabolic pathways.

Our second validation method involved the cellobiose-grown mixed-soil communities, for which we calculated Spearman correlation coefficients between predicted KO density and CUE. Those genes for which the approximate *t* statistic had a *P* value of less than 0.05 for the Spearman rank correlation were retained. We considered markers of CUE from the isolates to be validated when they had the same significant direction of correlation in the isolate exploratory glucose data sets, exploratory plus validating isolate glucose data sets, and microcosm or other substrate data sets. When the validating data set confirmed the correlation between a genomic marker and CUE that was proposed by the exploratory data set, we examined residual plots for bias and normality for models. Model residuals were also examined to confirm removal of any phylogenetic signal using the phylosig() function in phytools v.0.6-60 (82). Proposed markers which did not meet these criteria were excluded from further analysis, which in practice meant that “rare” functions found in just a few genomes were routinely removed.

### Inferring CUE based on phylogeny.

The overall phylogenetic signal for CUE was calculated using the phytools package (82) for both Blomberg’s *K* (83) and Pagel’s lambda (84). We then used the rescale function in geiger (85) to scale the terminal branch lengths of the phylogeny according to this lambda such the trait matched Brownian motion. Ancestral reconstruction of CUE and of its temperature sensitivity was completed on the rescaled tree using the phyEstimate() function in picante (86), where one known tip was removed at a time and the CUE of the remaining tips was used to infer that of the removed tip.

### Data availability.

All data and code required to reproduce the analysis presented in the manuscript, as well as the supplementary methods, are available in the Open Science Framework (OSF) (osf.io/ahb2v).
