# Supplementary material for: Carbon Use Efficiency and Its Temperature Sensitivity Covary in Soil Bacteria
Source: mBio. 2020 Jan 21;11(1):e02293-19. doi: 10.1128/mBio.02293-19 (PMC6974560; doi:10.1128/mBio.02293-19)
Supplement: FIG S1 [file mBio.02293-19-sf001.pdf]

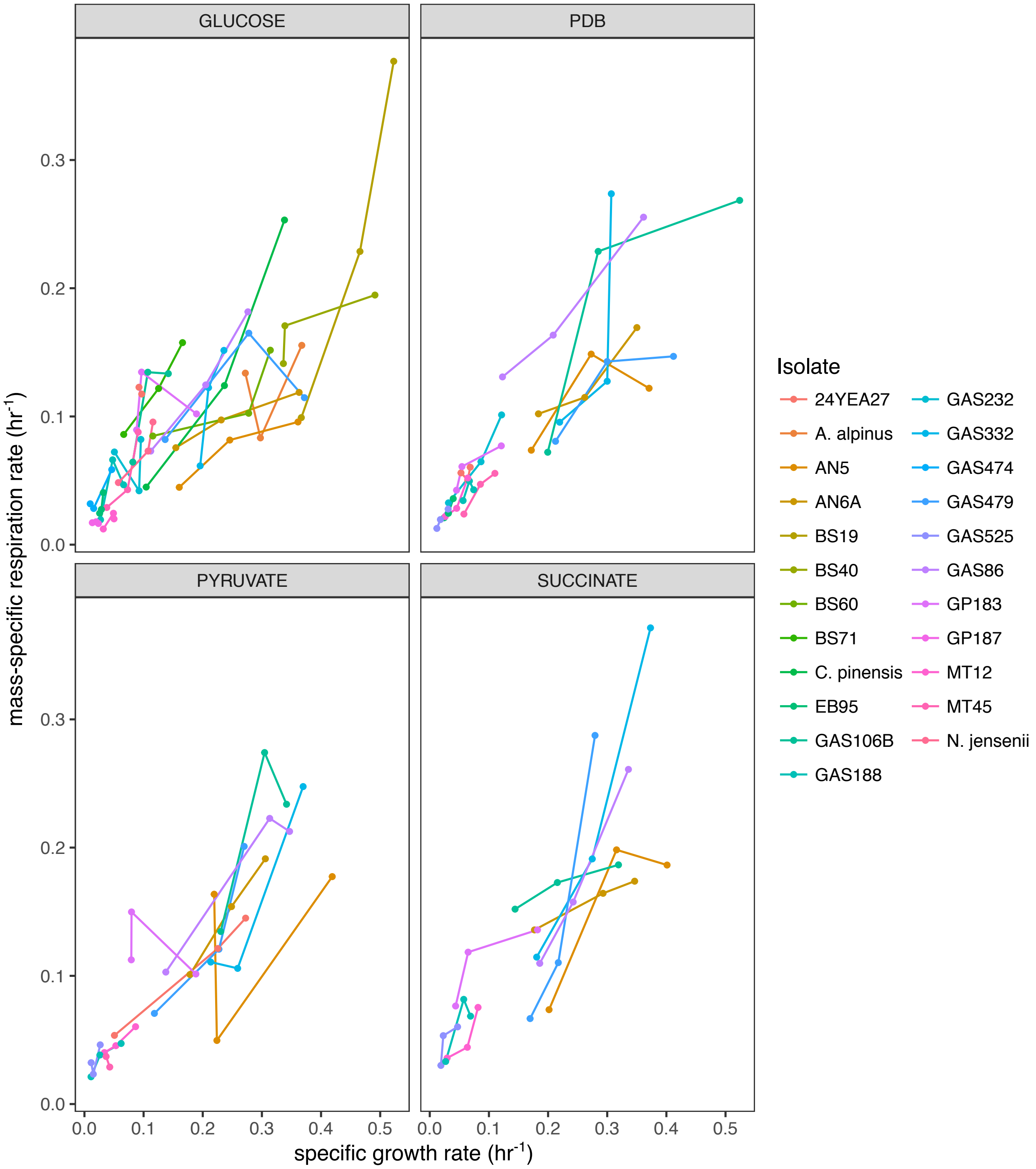

Figure S1: Responses of mass-specific respiration and growth rate to changes in substrate and temperature. Lines are coloured by isolate, such that a point represents the mean respiration rate and growth rate for a given temperature and substrate for an isolate. Lines are drawn to connect points corresponding to a given isolate, and do not imply a statistical relationship.
