# Supplementary material for: Carbon Use Efficiency and Its Temperature Sensitivity Covary in Soil Bacteria
Source: mBio. 2020 Jan 21;11(1):e02293-19. doi: 10.1128/mBio.02293-19 (PMC6974560; doi:10.1128/mBio.02293-19)
Supplement: FIG S2 [file mBio.02293-19-sf002.pdf]

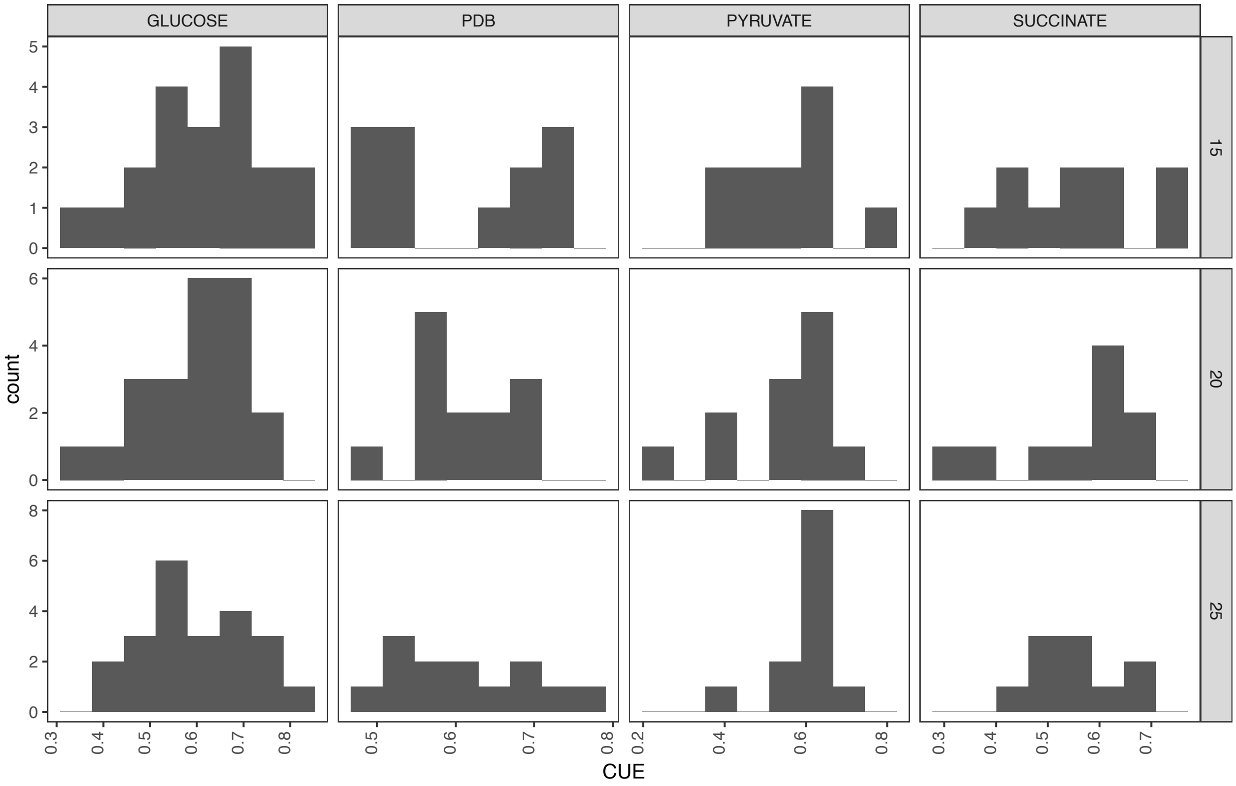

Figure S2: Frequency histograms of CUE of isolates grown on the four substrates at three temperatures. Each count is the average of all replicates for a given isolate under that assay condition.
