# Supplementary material for: Carbon Use Efficiency and Its Temperature Sensitivity Covary in Soil Bacteria
Source: mBio. 2020 Jan 21;11(1):e02293-19. doi: 10.1128/mBio.02293-19 (PMC6974560; doi:10.1128/mBio.02293-19)
Supplement: FIG S3 [file mBio.02293-19-sf003.pdf]

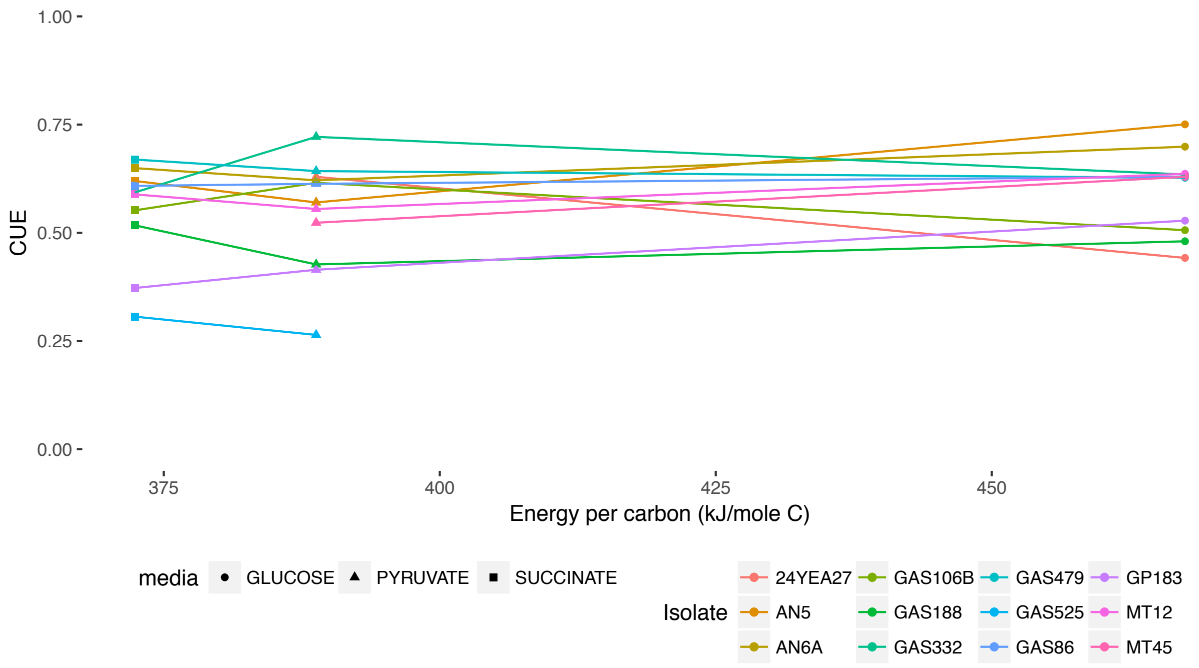

Figure S3: Effect of C quality on CUE. Each line denotes values for a different isolate. The x-axis is the heat of combustion of the substrate in kilojoules per mole divided by the number of C atoms in a mole of the substrate. Only cultures grown at 20°C are plotted
