# Supplementary material for: Carbon Use Efficiency and Its Temperature Sensitivity Covary in Soil Bacteria
Source: mBio. 2020 Jan 21;11(1):e02293-19. doi: 10.1128/mBio.02293-19 (PMC6974560; doi:10.1128/mBio.02293-19)
Supplement: FIG S4 [file mBio.02293-19-sf004.pdf]

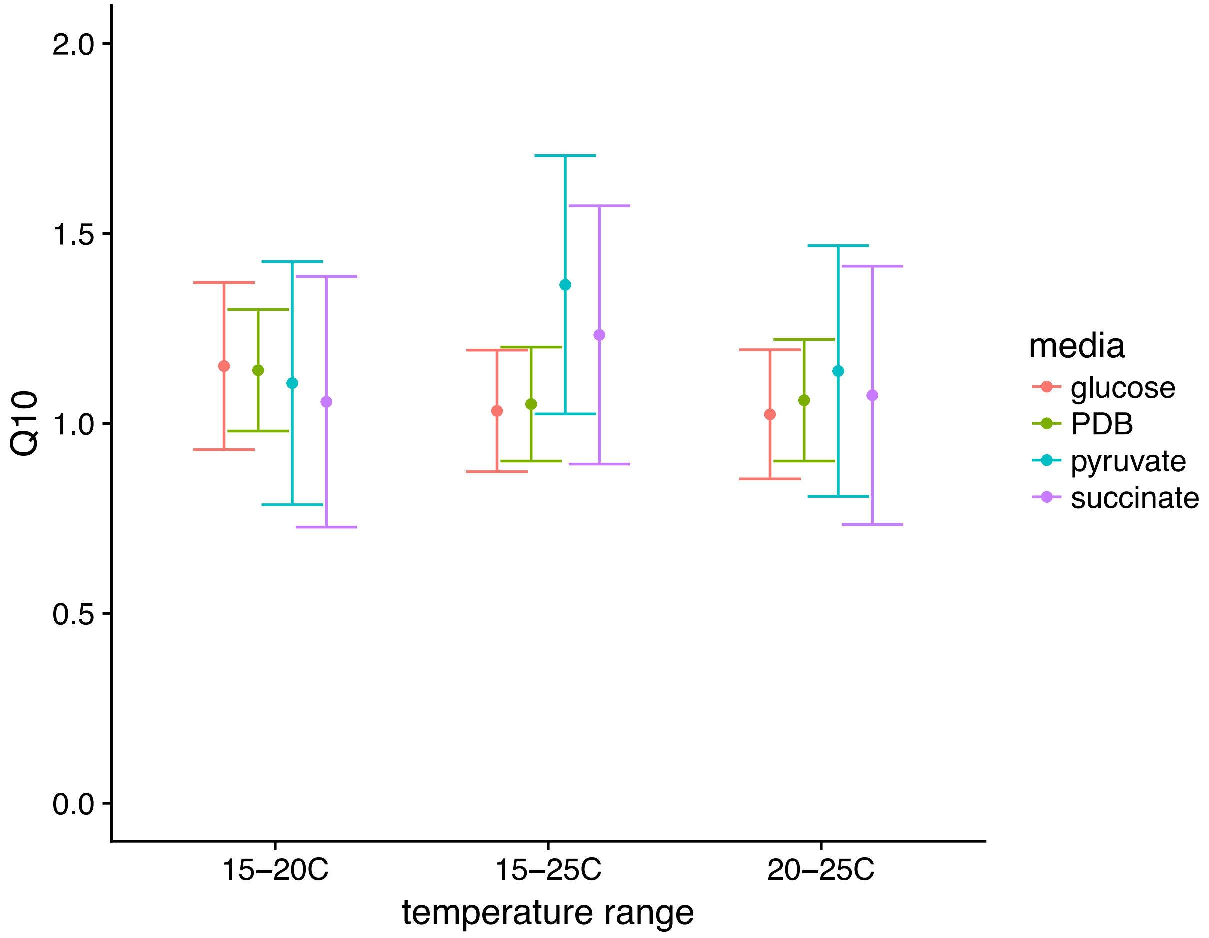

Figure S4: Phylogenetically-weighted mean temperature sensitivity of CUE for the four substrates and three temperature ranges used in this study, reported with 95% confidence intervals. Reported means and confidence intervals are the posterior estimates resulting from running an animal model in MCMCglmm.
