# Supplementary material for: Carbon Use Efficiency and Its Temperature Sensitivity Covary in Soil Bacteria
Source: mBio. 2020 Jan 21;11(1):e02293-19. doi: 10.1128/mBio.02293-19 (PMC6974560; doi:10.1128/mBio.02293-19)
Supplement: FIG S5 [file mBio.02293-19-sf005.pdf]

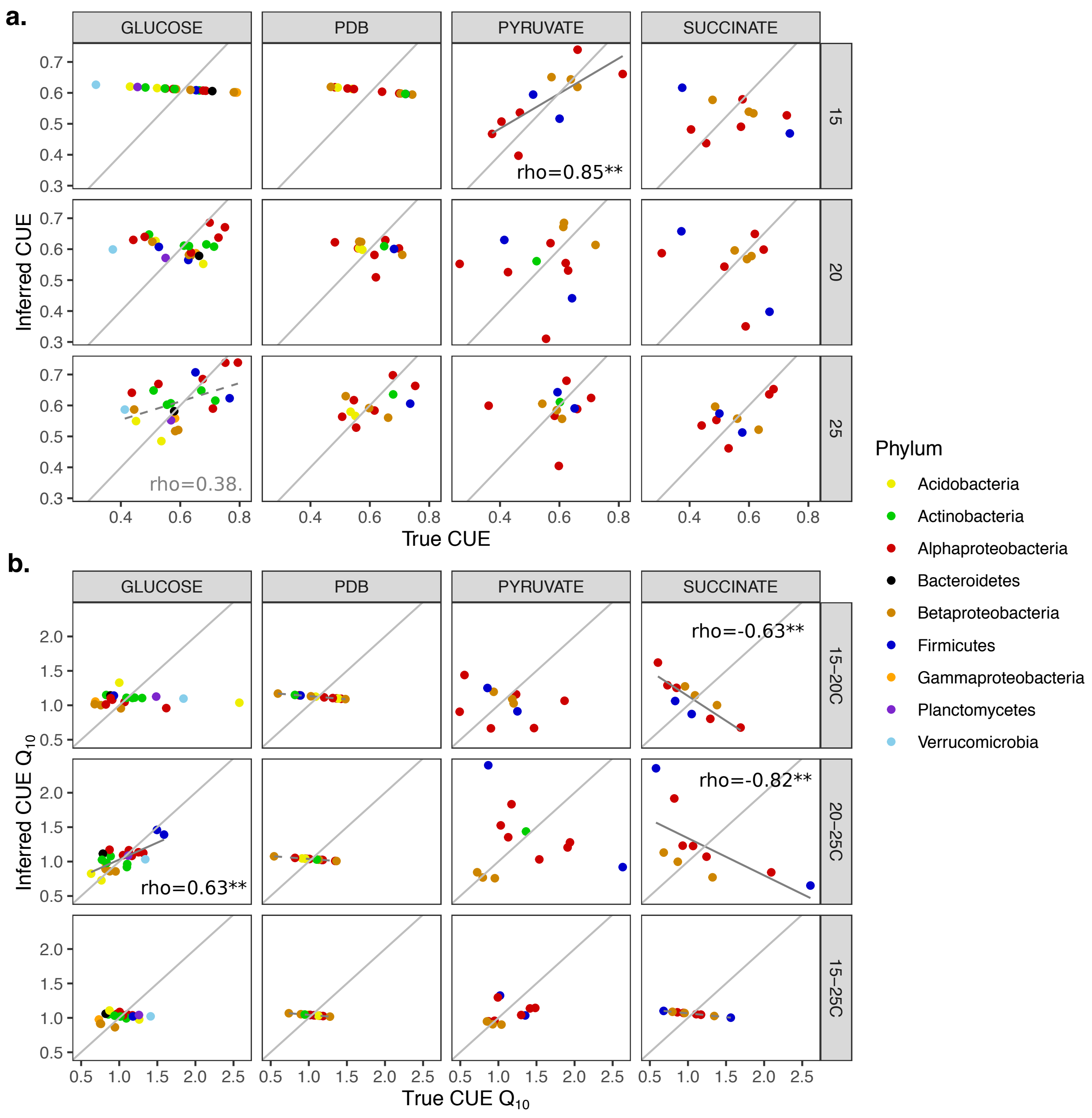

Figure S5: Plot of observed mean CUE (A) or CUE $Q_($B) for each isolate and incubation condition vs. the predicted mean CUE based on phylogenetic reconstruction using ancestral reconstruction techniques. Each point represents an isolate, the x-axis the observed mean CUE, and the y-axis the mean CUE predicted for the isolate based on ancestral reconstruction. The 1:1 line, indicating perfect agreement between predicted and observed CUE, is drawn in solid grey, and the correlation for significant relationships between observed and predicted mean CUE for each isolate is drawn as a heavier line. Spearman's rho is presented for those correlations ** $P < 0.01$,* $P < 0.05$, and . P <0.1
