# Supplementary material for: Carbon Use Efficiency and Its Temperature Sensitivity Covary in Soil Bacteria
Source: mBio. 2020 Jan 21;11(1):e02293-19. doi: 10.1128/mBio.02293-19 (PMC6974560; doi:10.1128/mBio.02293-19)
Supplement: FIG S6 [file mBio.02293-19-sf006.pdf]

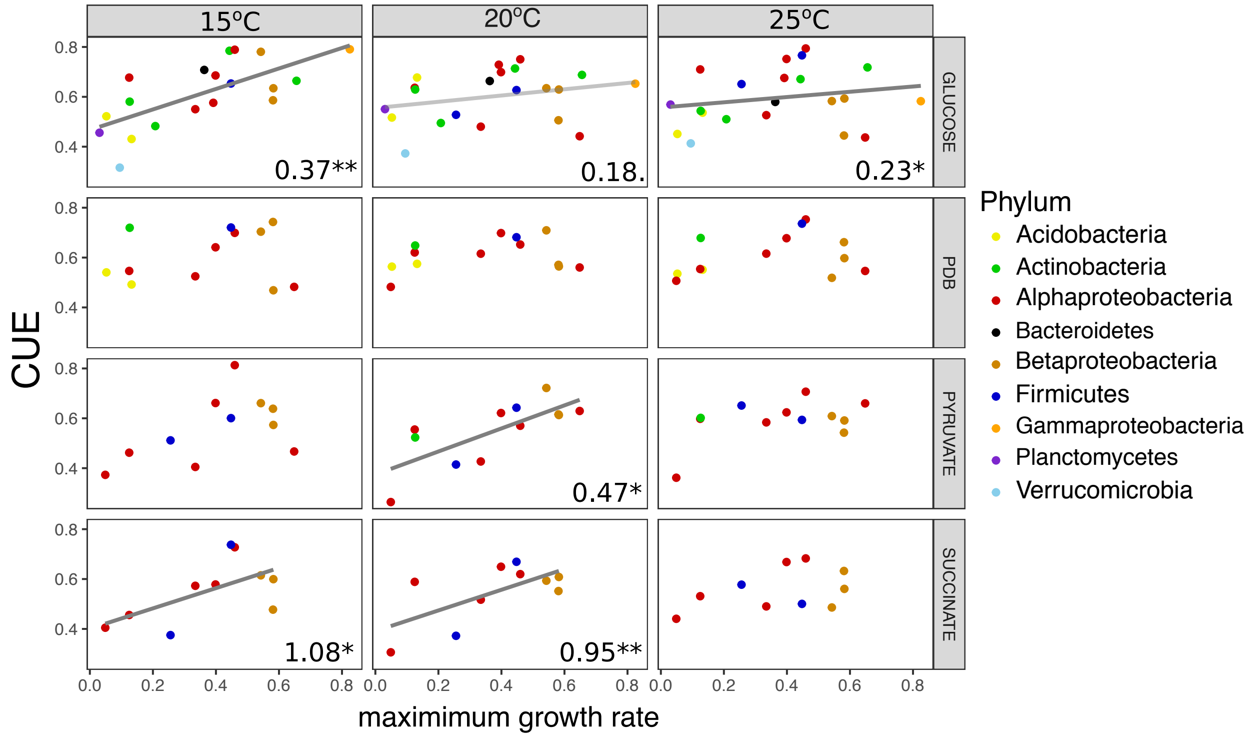

Figure S6: Correlation between CUE and maximum growth rate of taxa across the four substrates and three temperatures assayed. PGLS slopes are drawn, with numbers on each panel denoting the slope and its significance (** $P < 0.01$;* $P < 0.05$; . $P < 0.1$
