# Supplementary material for: Carbon Use Efficiency and Its Temperature Sensitivity Covary in Soil Bacteria
Source: mBio. 2020 Jan 21;11(1):e02293-19. doi: 10.1128/mBio.02293-19 (PMC6974560; doi:10.1128/mBio.02293-19)
Supplement: FIG S7 [file mBio.02293-19-sf007.pdf]

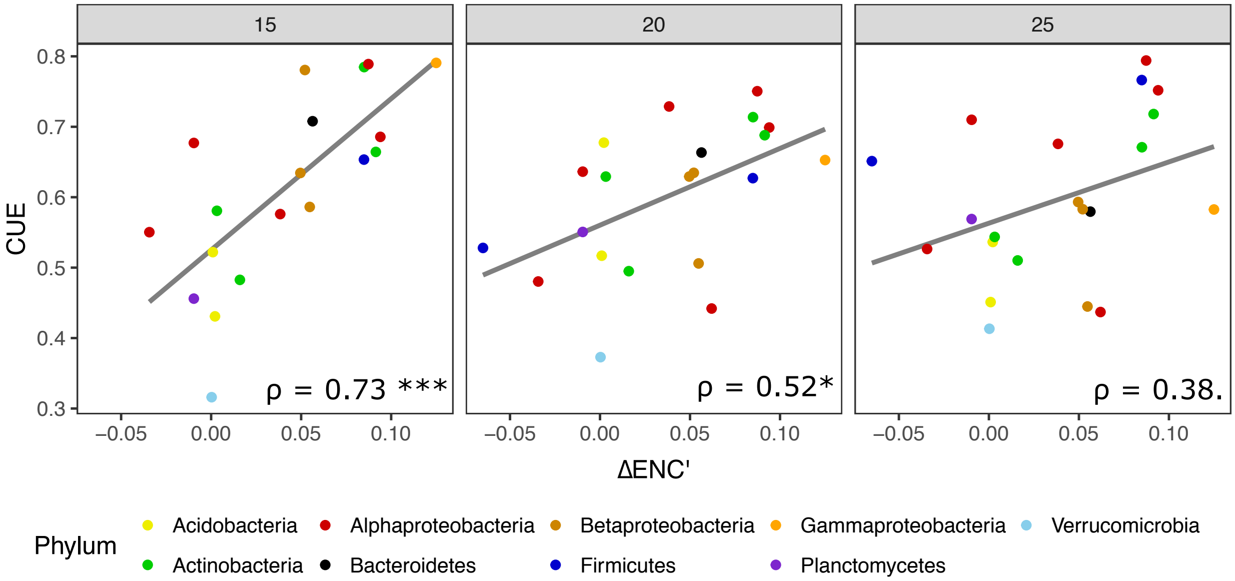

Figure S7: Correlations between CUE and codon bias (as per (41)) for bacterial isolates grown on glucose. Spearman correlation coefficients (not corrected for phylogenetic correlation) are included, along with the p-value (*** $P < 0.001$;* $P < 0.05$; . $P < 0.1$
