# Supplementary material for: Carbon Use Efficiency and Its Temperature Sensitivity Covary in Soil Bacteria
Source: mBio. 2020 Jan 21;11(1):e02293-19. doi: 10.1128/mBio.02293-19 (PMC6974560; doi:10.1128/mBio.02293-19)
Supplement: FIG S8 [file mBio.02293-19-sf008.pdf]

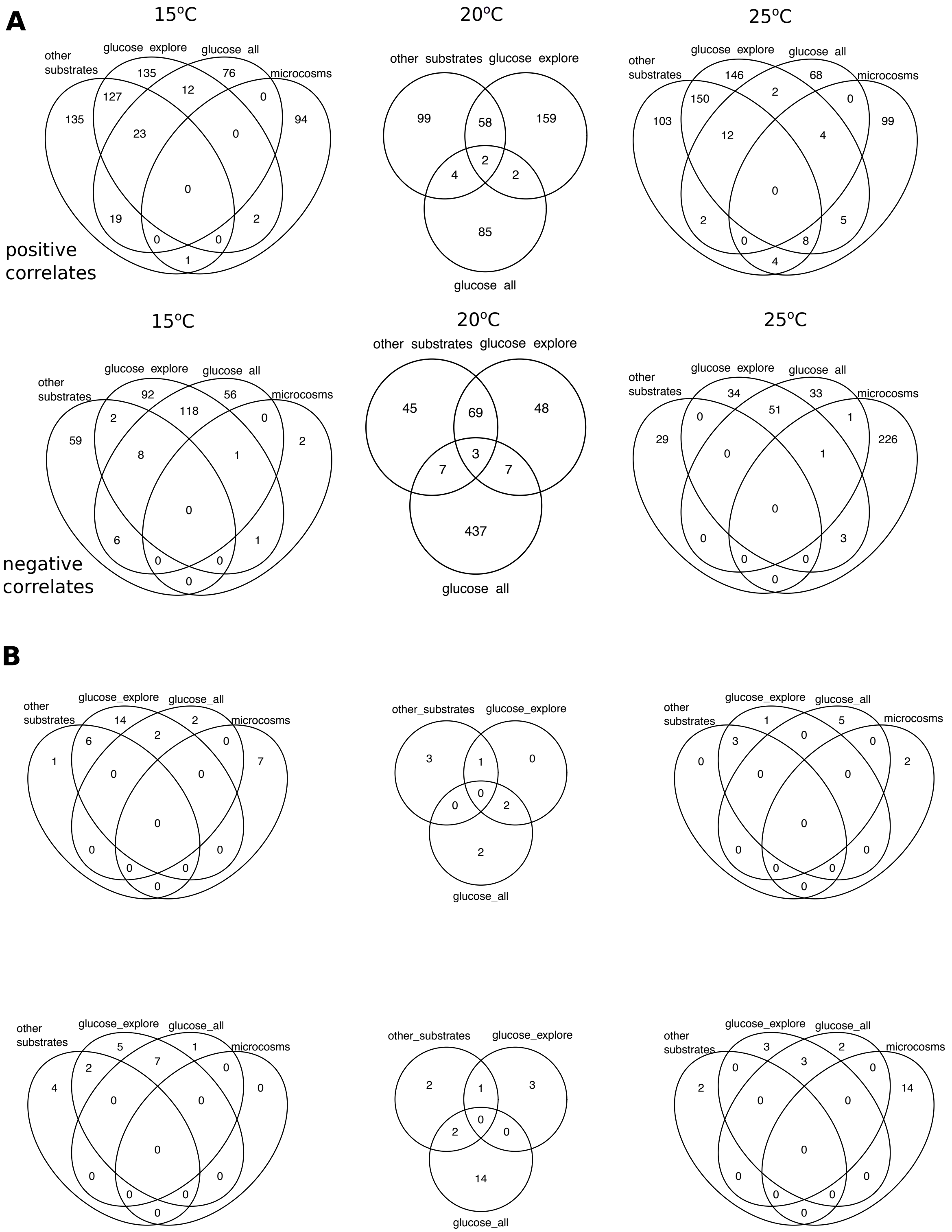

Figure S8: Venn diagrams of numbers of individual KO markers (A) or pathways (B) for which their genomic density is positively or negatively correlated with CUE. In all instances, “glucose explore” is considered to consist of the proposed markers of efficiency, while the remaining three datasets are considered as “validating” datasets. Sample sizes (number of isolates or microcosms) for each analysis are as follows: other substrates (10-13); glucose explore (13); glucose all (22); microcosms (10).
