# Supplementary material for: Carbon Use Efficiency and Its Temperature Sensitivity Covary in Soil Bacteria
Source: mBio. 2020 Jan 21;11(1):e02293-19. doi: 10.1128/mBio.02293-19 (PMC6974560; doi:10.1128/mBio.02293-19)
Supplement: TABLE S1 [file mBio.02293-19-st001.pdf]

	CUE vs. GRmax	CUE vs. rrN	CUE vs. $\log_2$ rrN	Metabolic pathway count	CUE for EEA production
15-20°C glucose	-	-	-	-0.069 .	-
20-25°C glucose	-	-	-	-	-
15-25°C glucose	-	-	-	-	-2.71*
15-20°C pyruvate	-	-	-	-	NA
20-25°C pyruvate	-	-	-	-0.021 .	NA
15-25°C pyruvate	-	-0.068 .	-	-0.010*	NA
15-20°C succinate	-	-	-	-	NA
20-25°C succinate	-	-	-	-0.051 **	NA
15-25°C succinate	-	-	-	-0.022*	NA
15-20°C PDB	-	-0.071 .	-	-	NA
20-25°C PDB	-	-	-	-	NA
15-25°C PDB	-	-	-	-	NA

Table S1: Regression coefficients for a phylogenetic generalized least squares model fit to CUE at a given substrate and temperature range versus ribosomal RNA operon copy number or the maximum growth rate observed across all assay conditions. Slopes are shown when the p-value is less than 0.1 (.), 0.05 (*), or 0.01 (**). Metabolic pathway count corresponds to the number of MAPLE pathways with $> 80\%$ completeness. CUE for EEA production corresponds to the theoretical fraction of C from glucose expected to be retained in the extracellular enzymes produced by the organism, rather than being burned to produce the ATP needed to make the corresponding amino acids de novo and then polymerize them into the proteins.
