# Supplementary material for: Carbon Use Efficiency and Its Temperature Sensitivity Covary in Soil Bacteria
Source: mBio. 2020 Jan 21;11(1):e02293-19. doi: 10.1128/mBio.02293-19 (PMC6974560; doi:10.1128/mBio.02293-19)
Supplement: TABLE S2 [file mBio.02293-19-st002.pdf]

Table S2: Genomic markers of CUE or CUE temperature sensitivity (Q_{10}) identified in exploratory and complete glucose datasets and confirmed by their presence as correlates of efficiency in the microcosms dataset or for at least two of the three remaining substrates in the liquid culture assays. Markers are included when the slopes were either both positive or both negative for the two datasets at an alpha of 0.05.

validation method	Temperature/ temperature range (°C)	corelation	KO
microcosms	15	negative	K09474 - acid phosphatase (class A)<br>K01625 - 2-dehydro-3-deoxyphosphogluconate aldolase / (4S)-4-hydroxy-2-oxoglutarate aldolase
	25	negative	K00809 - deoxyhypusine synthase
		positive	K02167 - TetR/AcrR family transcriptional regulator, transcriptional repressor of bet genes<br>K09815 - zinc transport system substrate-binding protein
other substrates	15	negative	K01733 - threonine synthase<br>K07095 - uncharacterized protein<br>K14441 - ribosomal protein S12 methylthiotransferase
		positive	K01752 - L-serine dehydratase<br>K02016 - iron complex transport system substrate-binding protein<br>K02072 - D-methionine transport system permease protein<br>K03786 - 3-dehydroquinate dehydratase II<br>K03825 - putative acetyltransferase<br>K05596 - LysR family transcriptional regulator, chromosome initiation inhibitor<br>K09780 - Uncharacterized protein<br>K12256 - putrescine—pyruvate transaminase
	20	negative	K14441 - ribosomal protein S12 methylthiotransferase
		positive	K03442 - small conductance mechanosensitive channel
	25	positive	K00864 - glycerol kinase<br>K02013 - iron complex transport system ATP-binding protein<br>K02015 - iron complex transport system permease protein<br>K02342 - DNA polymerase III subunit epsilon<br>K05846 - osmoprotectant transport system permease protein
microcosms	15-20	negative	K01638 - malate synthase [EC:2.3.3.9]<br>K07222 - putative flavoprotein involved in K+ transport
		positive	K02536 - UDP-3-O-[3-hydroxymyristoyl] glucosamine N-acyltransferase [EC:2.3.1.191]<br>K03560 - biopolymer transport protein TolR<br>K06168 - tRNA-2-methylthio-N6-dimethylallyladenosine synthase [EC:2.8.4.3]<br>K07023 - putative hydrolases of HD superfamily<br>K08191 - MFS transporter, ACS family, hexuronate transporter<br>K09747 - uncharacterized protein<br>K11085 - ATP-binding cassette, subfamily B, bacterial MsbA [EC:3.6.3.-]
	15-25	negative	K01638 - malate synthase [EC:2.3.3.9]<br>K02283 - pilus assembly protein CpaF
	20-25	negative	K00389 - putative membrane protein<br>K00508 - linoleoyl-CoA desaturase [EC:1.14.19.3]<br>K01858 - myo-inositol-1-phosphate synthase [EC:5.5.1.4]<br>K09764 - uncharacterized protein
		positive	K02049 - NitT/TauT family transport system ATP-binding protein<br>K07240 - chromate transporter
other substrates	15-20	positive	K08151 - MFS transporter, DHA1 family, tetracycline resistance protein
	20-25		K03417 - methylisocitrate lyase [EC:4.1.3.30]<br>K19294 - alginate O-acetyltransferase complex protein AlgI
